# Supplementary material for: Global perturbation of organic carbon cycling by river damming
Source: Nat Commun. 2017 May 17;8:15347. doi: 10.1038/ncomms15347 (PMC5442313; doi:10.1038/ncomms15347)
Supplement: Supplementary Information — Supplementary Figures, Supplementary Tables and Supplementary References [file ncomms15347-s1.pdf]

Supplementary Table 1: Probability distribution functions (PDFs) used in the Monte Carlo analysis. Note that parameters a and b are unrelated to those used in Equations 8, 9 and 12, 13.

Parameter	Range, units	PDF equation	PDF parameters	Ref.
Volume (V)	0.001-180 km^3	Pareto: $y = \left(\frac{1}{\sigma}\right)\left(1 + k\frac{x-\theta}{\sigma}\right)^{-1-\frac{1}{k}}$	σ= 0.0556487, k = 1.39388, θ = 0	[1]
Discharge (Q)	0.01-150 $km^3\ yr^{-1}$	Pareto: $y = \left(\frac{1}{\sigma}\right)\left(1 + k\frac{x-\theta}{\sigma}\right)^{-1-\frac{1}{k}}$	σ= 0.0511971, k = 2.12464, θ = 0	[1]
Latitude	-90.00 – 90.00°	Normal: $y = \frac{1}{\sigma\sqrt{2\pi}}e^{\frac{-(x-\mu)^2}{2\sigma^2}}$	σ = 35.5964, μ = 12.1614	[1]
Altitude	-33 – 4515 m	Lognormal: $y = \frac{1}{x\sigma\sqrt{2\pi}}exp\left\{\frac{-(\ln x-\mu)^2}{2\sigma^2}\right\}$	σ = 1.11179, μ = 5.71949	[1]
Inflow DOC concentration	0.001 – 1x10^5 ppm	Gamma: $y = \frac{1}{b^a\Gamma(a)}x^{a-1}e^{\frac{-x}{b}}$	a = 1.218, b= 5.886	[2]
Inflow POC concentration	0.1 – 10 000 µM	Pareto: $y = \left(\frac{1}{\sigma}\right)\left(1 + k\frac{x-\theta}{\sigma}\right)^{-1-\frac{1}{k}}$	σ= 0.991719, k = 208.827, θ = 0	[1,3]
Half-saturation constant (K_s)	2.0x10^5 – 6.3x10^6 mol TDP km^{-3}	Uniform	N/A	[4-12]
k_{bur}	1 – 15 yr^{-1}	Uniform	N/A	[13-17]
k_{20}	0.256 – 1.825 yr^{-1}	Normal: $y = \frac{1}{\sigma\sqrt{2\pi}}e^{\frac{-(x-\mu)^2}{2\sigma^2}}$	σ = 0.685027, μ = 0.174299	[18-20]
Autochthonous k_{20} scaling factor	1 – 6 (unitless)	Normal: $y = \frac{1}{\sigma\sqrt{2\pi}}e^{\frac{-(x-\mu)^2}{2\sigma^2}}$	σ = 1, μ = 3	[17,20,21]

Supplementary Table 2: Parameters used to fit Equations 8, 9 and 12, 13 for all scenarios with statistically significant ($p<0.05$) R^2 values given.

Scenario	$POC_{bur,allo}$	$POC_{min,allo}$	$DOC_{min,allo}$	$POC_{bur,auto}$	$TOC_{min,auto}$
1970, 2000	a = 0.8679	b = 0.1086	b = 1	a = 0.7505	b = 0.2428
	α = 8.401	β = 0.2728	β = 0.0391	α = 9.658	β = 0.3773
	R^2 = 0.82	R^2 = 0.25	R^2 = 0.74	R^2 = 0.60	R^2 = 0.25
2030 AM	a = 0.8635	b = 0.1119	b = 1	a = 0.7404	b = 0.2466
	α= 8.279	β = 0.2791	β = 0.04084	α = 9.995	β = 0.3728
	R^2 = 0.82	R^2 = 0.25	R^2 = 0.47	R^2 = 0.63	R^2 = 0.27
2030 GO	a = 0.8628	b = 0.1136	b = 1	a = 0.7417	b = 0.2463
	α = 8.511	β = 0.2648	β = 0.04121	α = 9.831	β = 0.3858
	R^2 = 0.82	R^2 = 0.26	R^2 = 0.34	R^2 = 0.62	R^2 = 0.25
2030 OS	a = 0.8672	b = 0.1089	b = 1	a = 0.7426	b = 0.2469
	α = 8.439	β = 0.2835	β = 0.03818	α = 10.05	β = 0.3667
	R^2 = 0.82	R^2 = 0.25	R^2 = 0.75	R^2 = 0.63	R^2 = 0.26
2030 TG	a = 0.8675	b = 0.1124	b = 1	a = 0.7402	b = 0.2471
	α = 8.273	β = 0.2547	β = 0.03804	α = 9.792	β = 0.4003
	R^2 = 0.82	R^2 = 0.25	R^2 = 0.80	R^2 = 0.62	R^2 = 0.25
2050 AM	a = 0.8593	b = 0.1180	b = 1	a = 0.7374	b = 0.2528
	α = 8.447	β = 0.2547	β= 0.03969	α = 9.881	β = 0.3802
	R^2 = 0.81	R^2 = 0.25	R^2 = 0.61	R^2 = 0.62	R^2 = 0.26
2050 GO	a = 0.8581	b = 0.1177	b = 1	a = 0.7375	b = 0.2523
	α = 8.390	β = 0.2803	β = 0.03998	α = 9.645	β = 0.3911
	R^2 = 0.81	R^2 = 0.25	R^2 = 0.71	R^2 = 0.63	R^2 = 0.26
2050 OS	a = 0.8605	b = 0.1167	b = 1	a = 0.7277	b = 0.261
	α = 8.405	β = 0.2687	β = 0.03978	α = 10.17	β = 0.3401
	R^2 = 0.82	R^2 = 0.27	R^2 = 0.60	R^2 = 0.59	R^2 = 0.26
2050 TG	a = 0.8626	b = 0.1135	b = 1	a = 0.7411	b = 0.2493
	α = 8.368	β = 0.2723	β = 0.04036	α = 9.569	β = 0.4009
	R^2 = 0.82	R^2 = 0.27	R^2 = 0.53	R^2 = 0.63	R^2 = 0.26

Supplementary Table 3: Estimated stream order and corresponding upstream catchment area.

Stream order	Upstream catchment area (km^2)
1	<4
2	<15
3	<60
4	<250
5	<1000
6	<4000
7	<16000
8	<63000
9	<250000
10	<$1x10^6$

Supplementary Table 4: Model sensitivity analysis for autochthonous OC. % change represents the differences in global burial and mineralization fluxes, relative to the values obtained with the default parameters. Sensitivity of parameters used to calculate P is discussed in the Methods, section Model sensitivity and uncertainty.

Parameter	Default value	Imposed change	Burial % change	Min % change
Age	40 years	Set to 10 years	<1%	<1%
Latitude	35.0°	±10%	±4%	±9%
Elevation	300m	±10%	<1%	<1%
k_{bur}	7 yr^{-1}	±10%	±5%	±5%
Initial TOC mass	0 mol	Set to $1x10^6$ mol	No effect	No effect
Temperature	19.6°C	+0.82°C	<1%	+2%
k_{20}	3 yr^{-1}	±10%	±3%	±8%

Supplementary Table 5: Model sensitivity analysis for allochthonous OC. % change represents the differences in global burial and mineralization fluxes, relative to the values obtained with the default parameters.

Parameter	Default value	Imposed change	Burial % change	DOC min % change	POC min % change
Age	40 years	Set to 10 years	No effect	No effect	No effect
Inflow POC concentration	10 μM	±10%	No effect	No effect	No effect
Inflow DOC concentration	5.71 ppm	±10%	No effect	No effect	No effect
Latitude	35.0°	±10%	±1%	±4%	±8%
Elevation	300m	±10%	<1%	<1%	<1%
k_{bur}	7 yr^{-1}	±10%	±3%	No effect	±6%
k_{hyd} (hydrolysis rate constant)	0.1 yr^{-1}	±10%	<1%	No effect	<1%
Initial POC and DOC mass	0 mol	Both set to $1x10^6$ mol	No effect	No effect	No effect
Temperature	19.6°C	+0.82°C	<1%	+1%	+2%
k_{20}	1 yr^{-1}	±10%	±9%	±9%	±4%

Supplementary Table 6: Summary of fluxes predicted in the model, and relevant global damming parameters. Note that several database entries have been removed in this analysis. These include Canadian oil sands tailings dams, barrages or diversion canals with no proper reservoirs, including the Farakka Barrage on the Ganges, and the five planned dams in Chilean Patagonia that have been cancelled.

Flux (Tmol yr^{-1}) or parameter	1970	2000	2030 GO	2030 AM	2030 TG	2030 OS	2050 GO	2050 AM	2050 TG	2050 OS
POC export to coast	11	12	11	12	11	11	11	11	11	11
DOC export to coast	13	14	14	14	14	14	14	14	14	14
POC load to watersheds	12	14	15	16	15	15	15	15	15	15
DOC load to watersheds	14	16	17	17	17	17	17	17	17	17
POC load to reservoirs	1.4-1.5	2.5	5.4	5.5	5.5	5.5	6.0	6.0	5.4	5.5
DOC load to reservoirs	2.3-2.4	4.1	9.4	6.8	9.5	6.9	7.6	7.6	9.3	9.0
Number of dams	3987-4393	6846	10547	10547	10547	10547	10547	10547	10547	10547
Total res volume (km^3)	3371-3573	6191	8503	8503	8503	8503	8503	8503	8503	8503
Dammed catchment area (10^7 km^2)	2.2– 2.4	3.5	4.7	4.7	4.7	4.7	4.7	4.7	4.7	4.7
Dammed % of total catchment area	17-18	27	36	36	36	36	36	36	36	36
Air temperature increase relative to year 2000	N/A	N/A	1.09	1.02	0.91	1.0	2.11	1.86	1.29	1.82
$POC_{bur,allo}$	0.75 – 0.81	1.7	3.3	3.5	3.4	3.4	3.3	3.3	3.2	3.4
$POC_{bur,auto}$	0.26-0.28	0.48	0.98	0.88	0.93	0.91	1.0	1.0	0.94	0.91
$POC_{bur,tot}$	1.0-1.1	2.2	4.3	4.4	4.3	4.3	4.3	4.3	4.1	4.3
$POC_{min,allo}$	0.08 – 0.09	0.27	0.37	0.39	0.37	0.38	0.40	0.38	0.36	0.40
$DOC_{min,allo}$	0.71 - 0.73	1.3	1.8	1.8	1.7	1.7	1.8	1.8	1.8	1.8
$TOC_{min,auto}$	0.08	0.14	0.30	0.26	0.29	0.27	0.32	0.32	0.30	0.29
$TOC_{min,flooded}$	2.4	0.74	2.7	2.7	2.7	2.7	0	0	0	0
$TOC_{min,tot}$	3.3	2.5	5.2	5.2	5.1	5.1	2.5	2.5	2.5	2.5
P	0.63-0.67	1.2	3.0	2.7	2.9	2.8	3.1	3.1	2.9	2.8
TOC load to watersheds + P	26-27	31	35	36	35	35	35	35	35	35
Total load eliminated	1.9-2.0	4.0	6.8	6.9	6.7	6.7	6.8	6.8	6.6	6.8
% eliminated	7	13	19	19	19	19	19	19	19	19

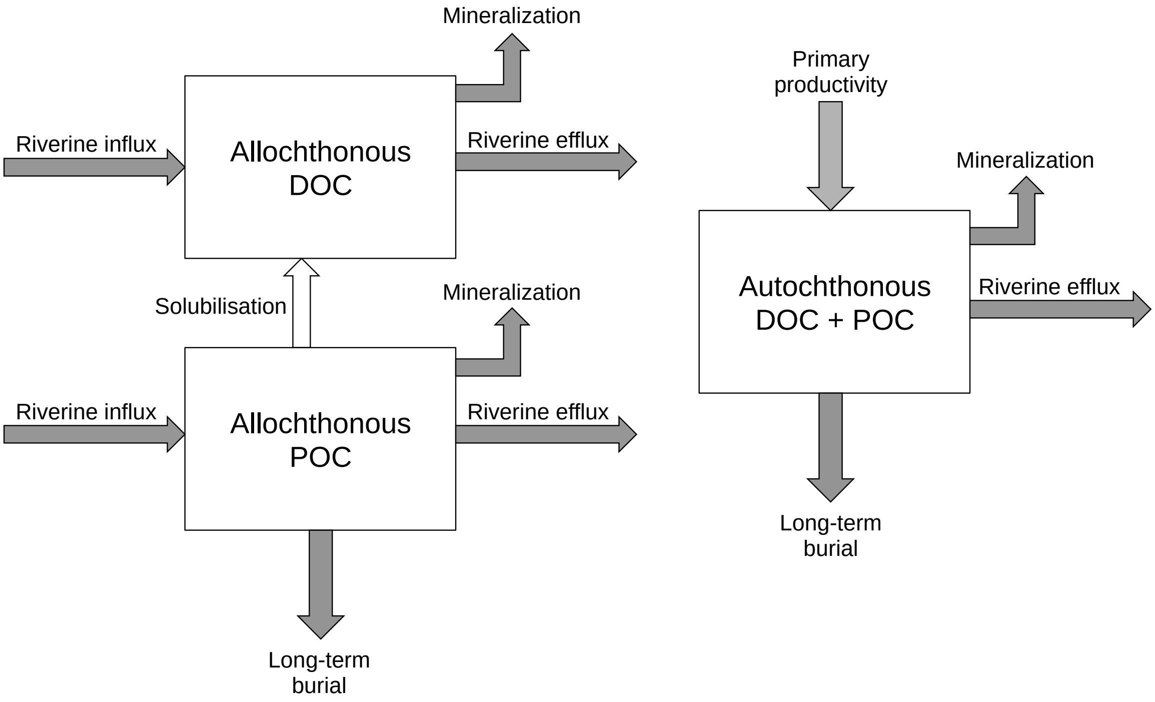

Supplementary Figure 1: Mechanistic model of in-reservoir organic carbon cycling.

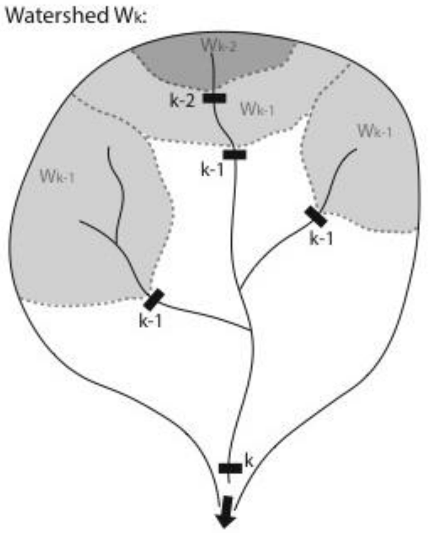

Supplementary Figure 2: Schematic representation of the breakdown of a hypothetical watershed into the sub-watersheds that are hydrologically connected to the dam reservoirs in the watershed; k represents the most downstream dam, k-1 the next dam upstream, and so on. The corresponding sub-watershed for dam k is W_k, W_{k-1} for dam k-1, and so on. The figure helps explain the routing procedure described in Methods section Global upscaling (see equation 10).

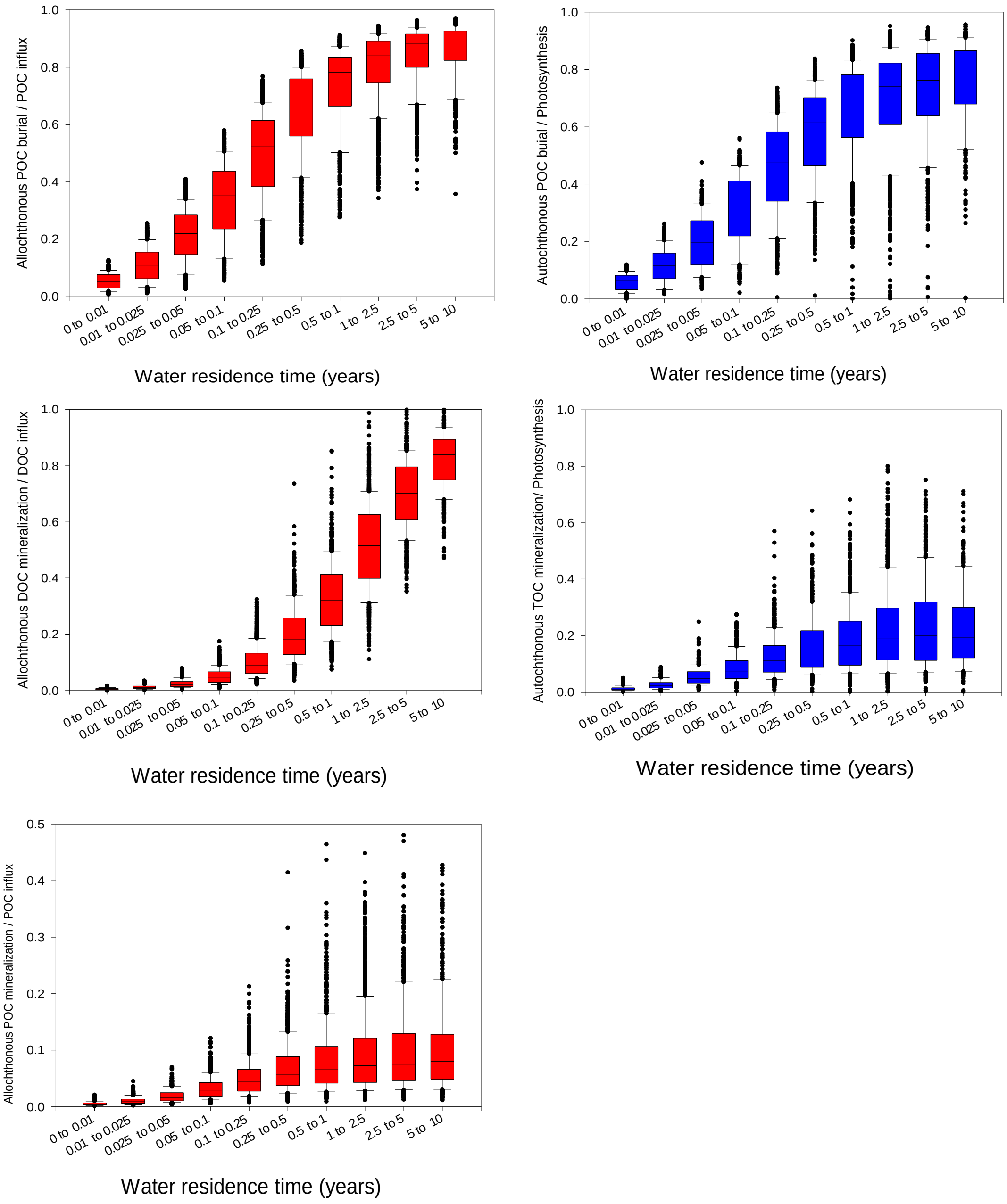

Supplementary Figure 3: Relative allochthonous and autochthonous burial and mineralization fluxes (normalized by *P* or POC or DOC influx) generated by the 6000 Monte Carlo iterations for years 1970 and 2000, and binned by water residence time. Solid lines represent median values, box edges represent 1 standard deviation and whiskers represent 1st and 3rd quartile.

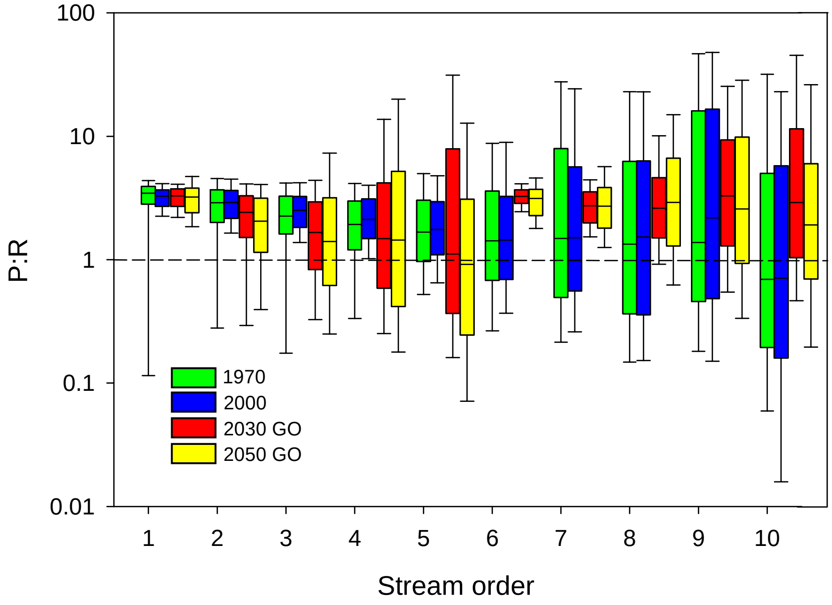

Supplementary Figure 4: Distributions of P:R ratios of reservoirs included in the GRanD and Zarfl et al.'s [22] databases as a function of Strahler stream order, for 1970, 2000, 2030 (GO scenario), and 2050 (GO scenario). Solid lines represent median values, box edges represent 1 standard deviation and whiskers represent 1st and 3rd quartile. For clarity, outliers have been removed.

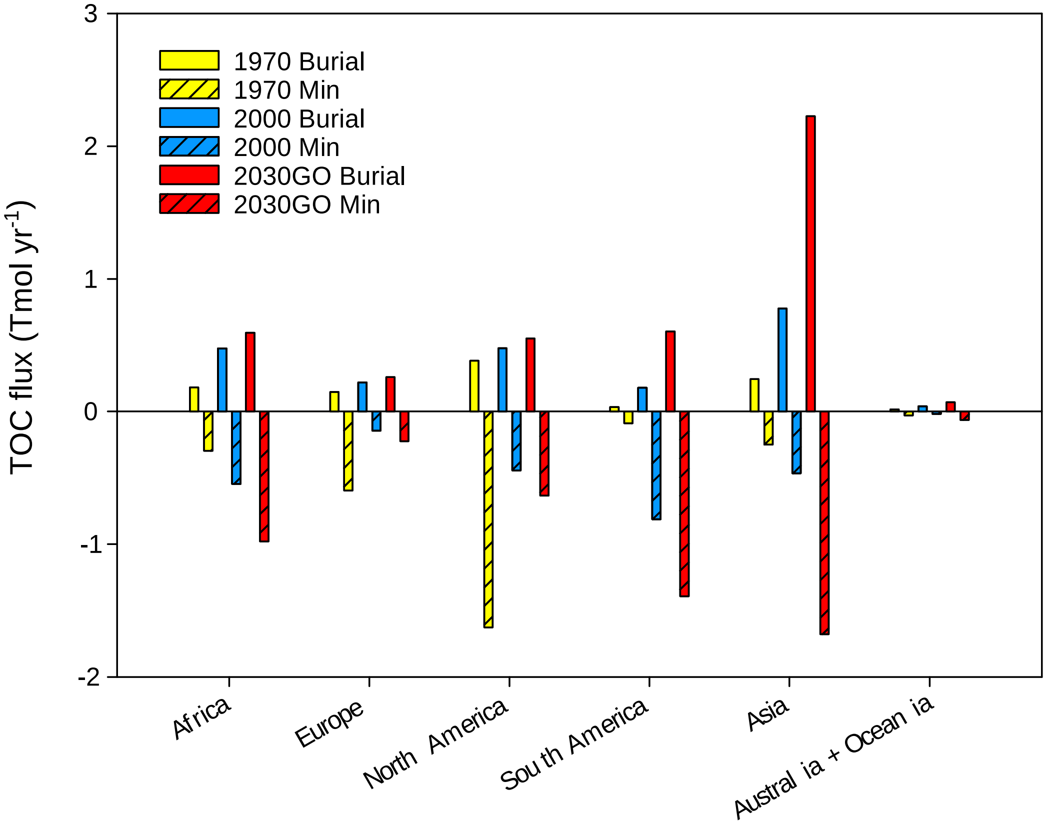

Supplementary Figure 5: Global OC burial and mineralization in reservoirs, for 1970, 2000, and 2030 (GO scenario). Mineralization fluxes are shown as negative values for clarity.

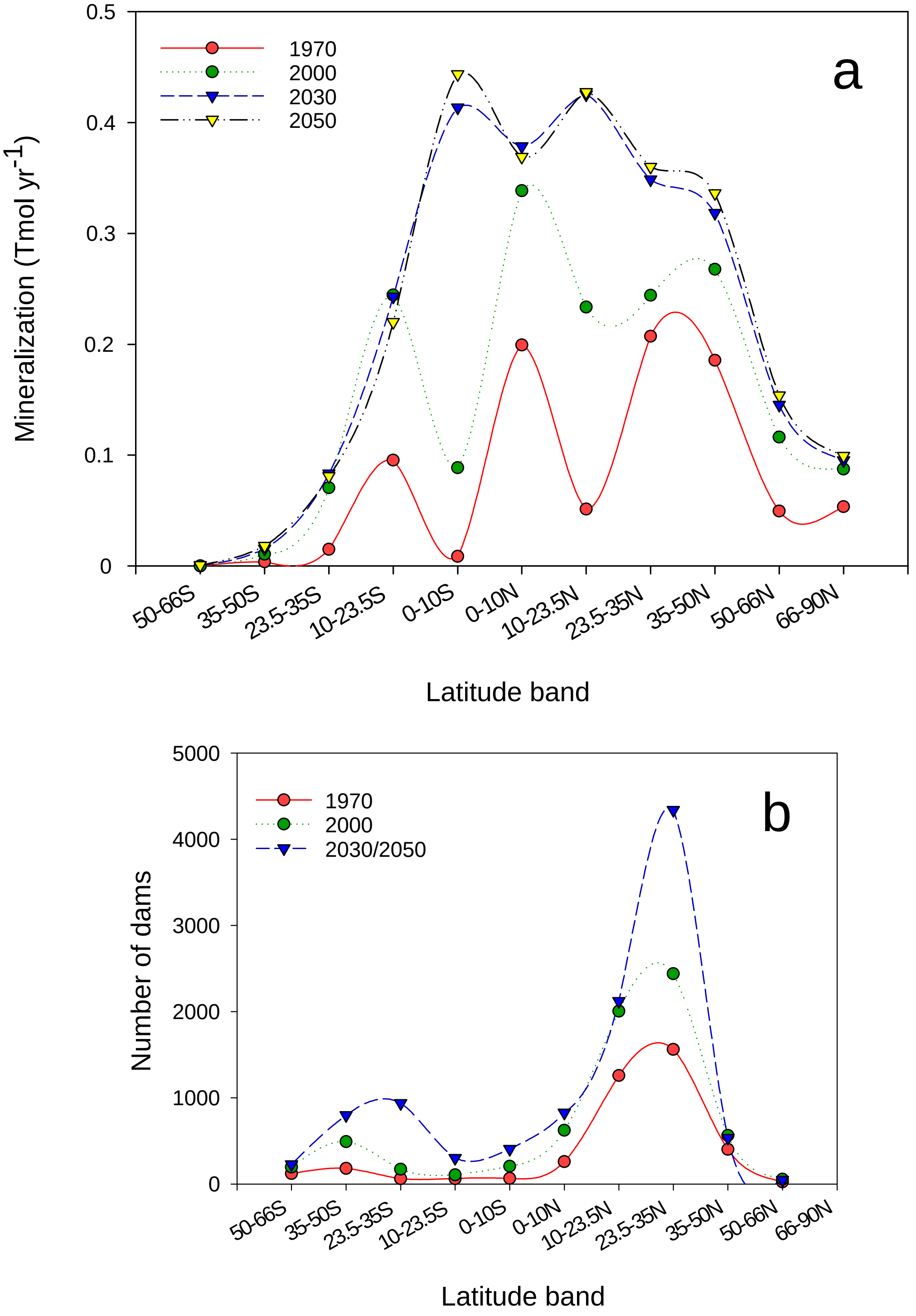

Supplementary Figure 6: (a) Reservoir OC mineralization fluxes by latitude band, excluding short-term degradation of flooded material, and (b) distribution of dams by latitude. Note that the number of dams in 2050 is assumed to be equal to that in 2030.

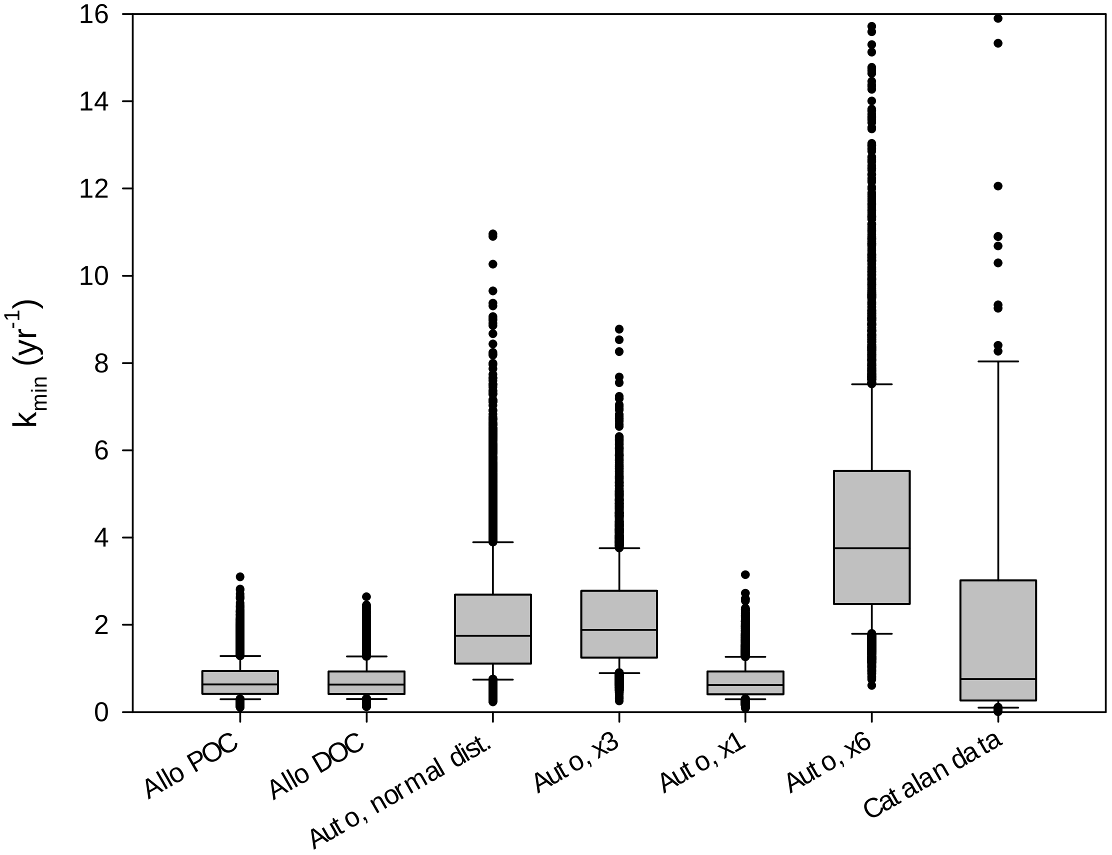

Supplementary Figure 7: Comparison between k_{min} values generated by the Monte Carlo (MC) procedure used in our model and the k_{min} values obtained independently by Catalan, et al. [20] from a global data compilation. The first three boxes to the left show the output of the MC analysis with the default model constraints (Supplementary Table 2). The boxes labelled "Auto, x3", "Auto, x1", and "Auto, x6" show additional outputs of MC analyses where the reactivity of autochthonous OC is assumed to be 3 times higher, equal, and 6 times higher than that of allochthonous OC, respectively. The last box shows the k_{min} distribution of Catalan et al., which lumps together values for POC and DOC, and for allochthonous and autochthonous OC. For clarity, extreme outliers of the Catalan data are not shown. Note that the default scaling factor of 3 used in the OC reservoir model (i.e., imposing a mean k_{min} value 3 times higher for autochthonous than allochthonous OC) is consistent with the observed k_{min} distribution of Catalan et al., while this is not the case for the lower (equal reactivity) or higher (6 times higher) scaling factors. Solid lines represent median values, box edges represent 1 standard deviation and whiskers represent 1st and 3rd quartile.

Supplementary References

1 Lehner, B. *et al.* High-resolution mapping of the world's reservoirs and dams for sustainable river-flow management. *Frontiers in Ecology and the Environment* **9**, 494-502, (2011).

2 Sobek, S., Tranvik, L. J., Prairie, Y. T., Kortelainen, P. & Cole, J. J. Patterns and regulation of dissolved organic carbon: An analysis of 7,500 widely distributed lakes. *Limnology and Oceanography* **52**, 1208-1219, (2007).

3 Mayorga, E. *et al.* Global Nutrient Export from WaterSheds 2 (NEWS 2): Model development and implementation. *Environ. Modell. Softw.* **25**, 837-853, (2010).

4 Reynolds, C. *Functional morphology and the adaptive strategies of freshwater phytoplankton*. (Cambridge University Press, 1988).

5 Nyholm, N. Kinetics of phosphorus-limited growth. *Biotechnology and Bioengineering* **19**, 467-472, (1977).

6 Tilman, D. & Kilham, S. S. Phosphate and silicate growth and uptake kinetics of the diatoms Asterionella formosa and Cyclotella meneghiniana in batch and semi-continuous culture. *Journal of Phycology* **12**, 375-383, (1976).

7 Lehman, J. T. Ecological and nutritional studies on Dinobryon Ehrenb.: Seasonal periodicity and the phosphate toxicity problem. *Limnology and Oceanography* **21**, 646-658, (1976).

8 Nalewajko, C. & Lean, D. in *Symposium: Experimental Use of Algal Cultures in Limnology 26-28 October 1976, Sandefjord, Norway. Internationale Vereinigung fur Theoretische und Angewandte Limnologie, Mittelungen.*

9 van Liere, L. & Mur, L. R. Growth kinetics of Oscillatoria agardhii Gomont in continuous culture, limited in its growth by the light energy supply. *Journal of General Microbiology* **115**, 153-160, (1979).

10 Ahlgren, G. Growth of oscillatoria agardhii in chemostat culture: 1. nitrogen and phosphorus requirements. *Oikos*, 209-224, (1977).

11 Ahlgren, G. in *Symposium: Experimental Use of Algal Cultures in Limnology 26-28 October 1976, Sandefjord, Norway. Internationale Vereinigung fur Theoretische und Angewandte Limnologie, Mittelungen.*

12 Holm, N. P. & Armstrong, D. E. Role of nutrient limitation and competition in controlling the populations of Asterionella formosa and Microcystis aeruginosa in semicontinuous culture. *Limnology and Oceanography* **26**, 622-634, (1981).

13 Dean, W. E. & Gorham, E. Magnitude and significance of carbon burial in lakes, reservoirs, and peatlands. *Geology* **26**, 535-538, (1998).

14 Jørgensen, S. E. A eutrophication model for a lake. *Ecological Modelling* **2**, 147-165, (1976).

15 Mulholland, P. J. & Elwood, J. W. The role of lake and reservoir sediments as sinks in the perturbed global carbon cycle. *Tellus* **34**, 490-499, (1982).

16 Kastowski, M., Hinderer, M. & Vecsei, A. Long‐term carbon burial in European lakes: Analysis and estimate. *Global Biogeochemical Cycles* **25**, (2011).

17 Sobek, S. *et al.* Organic carbon burial efficiency in lake sediments controlled by oxygen exposure time and sediment source. *Limnology and Oceanography* **54**, 2243-2254, (2009).
18 Hanson, P. C., Buffam, I., Rusak, J. A., Stanley, E. H. & Watras, C. Quantifying lake allochthonous organic carbon budgets using a simple equilibrium model. *Limnology and Oceanography* **59**, 167-181, (2014).
19 Hanson, P. C. *et al.* Fate of allochthonous dissolved organic carbon in lakes: a quantitative approach. *PloS one* **6**, e21884, (2011).
20 Catalan, N., Marce, R., Kothawala, D. N. & Tranvik, L. J. Organic carbon decomposition rates controlled by water retention time across inland waters. *Nature Geosci* **9**, 501-504, (2016).
21 Koehler, B., Wachenfeldt, E., Kothawala, D. & Tranvik, L. J. Reactivity continuum of dissolved organic carbon decomposition in lake water. *Journal of Geophysical Research: Biogeosciences (2005–2012)* **117**, (2012).
22 Zarfl, C., Lumsdon, A., Berlekamp, J., Tydecks, L. & Tockner, K. A global boom in hydropower dam construction. *Aquat. Sci.* **77**, 161-170, (2015).
